# Saccharide Characteristics and Their Potential Health Effects in Perspective

**DOI:** 10.3389/fnut.2020.00075

**Published:** 2020-07-06

**Authors:** Fred Brouns

**Affiliations:** Department of Human Biology, Faculty of Health, Medicine and Life Sciences, School of Nutrition and Translational Research in Metabolism, Maastricht University, Maastricht, Netherlands

**Keywords:** saccharide-characteristics, carbohydrate-classification, added sugars, free sugars, sugar-functionality, glycemic index, sugars and health

## Abstract

To understand the effects of saccharides on our metabolism and health, we need a clear understanding of what they are, how they differ, and why some types are deemed “less healthy” and others “better for health.” There are various ways to look at this topic. Firstly, saccharides can be classified according to their degree of polymerization (DP). This classification is useful when qualitative or quantitative analysis and calculation of intakes are required or for food-labeling definitions. However, it does not account for the fact that saccharides with a similar DP can differ in molecular composition, which will influence digestion, absorption, and metabolism. Secondly, another approach widely used in the biomedical and nutritional sciences is therefore a physiological classification, which addresses the rate and degree of digestibility and absorption, the glycemic response, and the metabolic fate. The individual health status also plays a role in this respect. An active, lean person will have a metabolic response that differs from an inactive person with overweight and insulin resistance. However, this approach will not give a complete answer either because the characteristics of the matrix/meal in which these carbohydrates (CHOs) are present will also influence the responses of our body. Thirdly, one can also rank CHOs by comparing their functional/technological properties, such as relative sweetness, viscosity, and solubility. Understanding CHO characteristics and related physiological responses will help understand health and disease implications. Therefore, a brief outline of different carbohydrate classifications is presented. This outline will be placed in the context of potential overall effects after consumption. The answer to the question whether we should we eat less of certain sugars depends on the angle from which you look at this matter; for example, do you address this question from a single molecular characteristic point of view or from a meal quality perspective? Looking at one particular CHO characteristic will almost always lead to a different conclusion (e.g., the labeling of fructose as toxic) than evaluating from a “total perspective” (fructose has adverse effects in certain conditions). Examples are given to help understand this matter for the benefit of justified dietary/food-based recommendations.

## Introduction

Sugars and other carbohydrates (CHOs) have many characteristics, ranging from molecular composition to functional, physiological, and biochemical behavior. As any individual characteristic of a given CHO can influence its physiological properties, it should be viewed in the context of all other characteristics. For example, different sugars can be similar with respect to their monomer composition but may differ in the bonds between these constituents. Ingesting sucrose, which delivers the monomers glucose and fructose for absorption, can lead to different gastrointestinal and post-absorptive effects compared with ingesting glucose or fructose as a single source. In a solid, liquid, or viscous matrix, the same sugars will show different physiological responses. For this reason, it needs to be acknowledged that looking at one particular CHO characteristic will almost always lead to a different conclusion about potential health effects than looking from a “total” perspective as regards the effect of the carbohydrate in a certain meal/pattern and lifestyle. A consequence may be that misinterpretations and misconceptions are being created by interpreting the effects of saccharides on health in a strongly reductionistic way (1–4). In this light, the reader will be provided with condensed information on specific compositional characteristics of CHOs, especially sugars, which have physiological and metabolic effects. The individual characteristics will be discussed in the context of what they mean for the potential overall effects on health and disease, and why food authorities are shifting to more qualitative food-based guidelines (5–8).

## Chemical Classification of Saccharides and Its Meaning

Saccharides can be ranked according to the characteristics of their molecular composition. This ranking includes individual monomers (monosaccharides) and the number of bonds. For example, sucrose is comprised of two monomers, glucose and fructose, which are linked by an α1,2 glycosidic bond, having a degree of polymerisation (DP) of 2. Chemical classifications that are commonly used by nutrition and food safety authorities (9) are as follows:

1. Sugars (monosaccharides and disaccharides, DP 1–2)
2. Oligosaccharides (DP 3–9)
3. Polysaccharides (DP ≥ 10).

Within these categories, dietary CHOs can be further subclassified as presented in Table 1 below.

**Table 1:** Chemical classification of carbohydrates (9–11) *Maltodextrins are an industrially hydrolyzed starch product.

**Classification**	**Sub-group**	**Examples**
Sugars (DP 1-2)	• Monosaccharides • Disaccharides • Sugars alcohols/polyols	• Glucose, fructose galactose, mannose, arabinose, xylose, erythrose, and others. • Sucrose, isomaltulose, lactose, maltose, trehalose, and others. • Sorbitol, mannitol, lactitol, xylitol, erythritol
Oligosaccharides (DP 3-9)	• Maltodextrins* (Malto-oligosaccharides) • Non-digestible oligosaccharides • Starch	• *Contain: glucose, maltose gluco-oligosaccharides • Raffinose, stachyose, fructo-oligosaccharides (FOS), arabino-oligosaccharides (AXOS), and others. • Amylose, amylopectin, and modified starches.
Polysaccharides (DP >9)	• Non-starch polysaccharides (NSP) • Resistant starch (RS)	• Pectin, cellulose, hemicellulose, hydrocolloids (Arabic gum, guar gum, others). • RS type 1,2,3, and 4

*During hydrolysis, a mixture of gluco-oligosaccharides, maltose, and glucose is formed. The quantity of glucose and maltose present in “maltodextrins” depends on the extent of hydrolysis (rate x time)*.

Dietary CHOs can be further subclassified as presented in Table 1 below.

### Same Degree of Polymerization but Different Effects

When present in disaccharides, the bonds of the composing monomers (α or β glycosidic bond) can differ, which will affect the rate of digestion and absorption. In Table 2, the chemical classifications and molecular characteristics of selected CHOs (types, bonds) are given, along with some selected characteristics of digestion, absorption, distribution, and metabolic fate.

**Table 2:** Chemical and physiological characteristics of sugars and other glycemic carbohydrates.

**CHO**	**Type**	**Digestive enzyme**	**In gut lumen**	**Enterocyte uptake**	**In blood**	**Possible metabolic fate options**	**GI**
Glucose	Monosaccharide	-	Glucose	-	Glucose	Used as fuel, stored as glycogen and/or converted to other metabolites	100
Fructose	Monosaccharide	-	Fructose	-	Lactate, glucose, fructose	Partially converted to lactic acid and glucose, used as fuel or stored as glycogen, and fatty acids used as fuel or triacylglycerol stored as lipid	19
Sucrose	Disaccharide: glucose -fructose, α1-2 bond	Sucrase	Glucose, fructose	Glucose, fructose	glucose, lactate, fructose	see glucose and fructose above	65
Isomaltulose	Disaccharide: glucose -fructose, α1-6 bond	Isomaltase	Glucose, fructose	Glucose, fructose		See fate of glucose and fructose above	32
Galactose	Monosaccharide	-	Galactose	-	Galactose	Liver conversion to glucose, see fate of glucose above	25
Lactose	Disaccharide: glucose -galactose, α1-4 bond	Lactase	Glucose, galactose	Glucose, galactose	Glucose, galactose	See fate of glucose and galactose above	45
Honey	Glucose 30.3%, fructose 38.4%, sucrose 1.3%	Sucrase	Glucose, fructose	Glucose, fructose	Glucose, lactate, fructose	See glucose and fructose above	50
Maple syrup	Sucrose 98%, glucose 1%, fructose 1%	Sucrase	Glucose, fructose	Glucose, fructose	Glucose, lactate, fructose	See glucose and fructose above	54
HFCS 55	Fructose 55%, glucose, 43% gluco-oligo saccharides 3%	α-Dextrinase	Glucose, fructose	Glucose, fructose	Glucose, lactate, fructose	See glucose and fructose above	58
Starch	Glucose polymers: amylopectin α1-4 and α1-6 bonds. Amylose α1-4 bonds	Amylase from saliva, pancreas	Maltose, glucose	Maltose, glucose	Glucose	See fate of glucose above	40–110*
Maltodextrins	Glucose polymer, α1-4 glycosidic bonds	α -Dextrinase	Glucose, maltose	Maltose, glucose	Glucose	See glucose above	110
Maltose	Disaccharide: glucose-glucose, α1-4 glycosidic bond	Maltase	Glucose	Glucose	Glucose	See glucose above	105
Trehalose	Disaccharide: glucose-glucose, α1-1 glycosidic bond	Trehalase	Glucose	Glucose	Glucose	See glucose above	70
Sorbitol*	Sugar alcohol	-	Sorbitol	-	Sorbitol	Liver conversion to fructose and glucose, see above	4

For a review of fructose, see Tappy and Lê (12). For a review of lactose and galactose, see University of Waterloo (13). One example of a low-caloric/low-glycemic sugar replacer is given. In the gut, sorbitol, a sugar-alcohol, is slowly absorbed (25–80% of the consumed dose) by facilitated diffusion. Absorbed sorbitol passes the liver, where it is converted to fructose and glucose (14). The unabsorbed fraction is transported to the large bowel, where it is fermented. When sorbitol is consumed in high doses, potential side effects can occur as a result of osmotic water shifts from blood into the gut, resulting in rumbling, loose stools, or diarrhea (extensive details about polyols can be found in Livesey (14), Ghosh and Sudha (15), Rice et al. (16). For a review of low- and non-caloric/non-glycemic sweeteners compared with caloric sweeteners, see Rogers et al. (17). *The glycemic index of starchy foods varies according to the molecular content of amylose, amylopectin, fiber, presence of protein, and characteristics of the food matrix, resulting in a range of reported values. For extensive glycemic index data [see (18)], International Tables of Glycemic Index and Glycemic Load Values, the online University of Sydney searchable data GI; http://www.glycemicindex.com/foodSearch.php. For further extensive details, see Queen Mary University London (19), Nomenclature of Carbohydrates (Available online at: https://www.qmul.ac.uk/sbcs/iupac/2carb/00n01.html#0121) and nomenclature of sugar alcohols (20).

To explain how CHOs with a similar monomer composition can differ in their degree of digestion and absorption, we will give two examples: [1] sucrose and isomaltulose, and [2] amylose and amylopectin starch.

**Figure 1:**
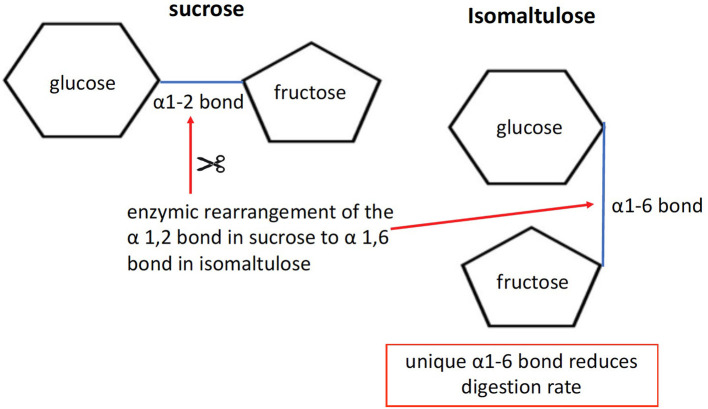
Molecular structure of isomaltulose. By using the microbial enzyme “glucosyl transferase” for rearranging the bond structure from α1-2 in sucrose, as base substrate, to α1-6, isomaltulose is formed.

**Figure 2:**
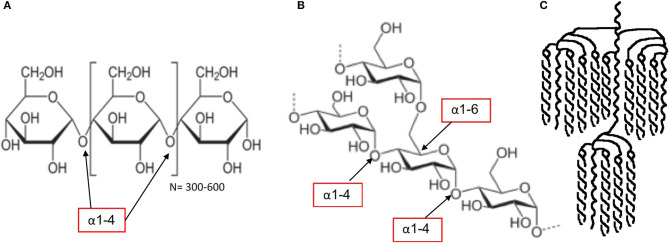
Molecular structure starch: amylose starch **(A)**, amylopectin starch **(B,C)** [figures source (24): starch, retrieved Jan 2020].

1. The disaccharides sucrose and isomaltulose are both composed of the two monomers glucose and fructose. However, the linkage between the two monomers differs. Sucrose has an α-1,2 bond, whereas isomaltulose has an α-1,6 bond (see Figure 1). Due to its more stable α-1,6 glycosidic bond, hydrolysis by small intestinal disaccharidases is slow. In human small intestinal mucosa homogenates as an enzyme source, the hydrolysis rate was 26–45% compared with sucrose (21). The result is a lower glycemic and insulinemic response (22), and consequently a reduced rate of metabolism (23).
2. One may wonder why the example of amylose and amylopectin starch is being discussed alongside sugars. The reason for including the example of starch is that sugars deliver their constituent monomers to the intestinal cells for absorption as a digestive fate. In the light of the generally accepted definition that sugars are all CHOs with a DP of 1–2, it becomes clear that both “sugar” and “starch” deliver “sugars” to the intestinal cells for absorption. In terms of metabolic responses, especially when comparing “sugars” with “starches,” it is good to have a clear comparative view. Plant starch generally contains 20–30% by weight of amylose and 70–80% by weight of amylopectin. Amylose (Figure 2A) contains linear chains of approximately 300–3,000 glucose monomers in length, connected by α-1,4 bonds. In amylopectin, there is also a linear basic structure in which glucose monomers are linked by α-1,4 bonds (Figures 2B,C), but there are side branches along this linear base initiated with α-1,6 bonds. This situation results in a molecule with many branching endpoints and a more open structure in which digestion enzymes can act, compared with the more closed linear helix formation of amylose. The digestive enzyme α-amylase is responsible for the breakdown of the starch into dextrins (maltotriose, DP3) and maltose (DP2), which are in turn digested by epithelial maltase, resulting in glucose monomers. It is often suggested that the amylose content is the most important factor in determining the rate of digestion and absorption as well as the related glycemic response, but recent research shows that the picture is more complex (25). It appears that the interaction between the molecular and granular structure (helix formation, number of pores, size of the molecule, amylopectin sidechain length distribution and crystalline structure, the latter two being the most important) causes the variation in the rate of digestion across botanical sources (25). The latter leads to relatively rapid digestion and a significant blood glucose response. The potential of starch to affect the blood glucose response, expressed as a glycemic index (GI) value, can therefore vary considerably depending on the content of amylopectin and amylose (26–28). Interestingly, despite only small differences in amylose content, *in vitro* cumulative starch hydrolysis shows that wheat starch is more rapidly digested than potato starch (being the most resistant starch), with corn, high-amylose corn, and pea starch having intermediate values (25).

Accordingly, rapidly digestible (available) CHOs, slowly digestible (available) CHOs, and non-digestible (non-available) CHOs (dietary fibers) can be ranked (18, 29–31). Along similar lines, digestible starch (glycemic) and resistant starch (not digested, non-glycemic) are both polysaccharides composed of glucose monomers and are both present in starchy foods, but they differ strongly in bioavailability. As a result, there is a wide range of GI values for different varieties of rice, cereals, potatoes and derived products, ranging from relatively low to high GI values (18). For this reason, one cannot establish a generic GI value for starchy foods. These aspects are important to understand for situations in which a rapid or sustained delivery of glucose to the circulation and tissues is required (e.g., sports nutrition or compensation of insulin dosage-induced hypoglycemia in diabetes patients), or generally to be avoided (type 2 diabetes).

In infant nutrition, sports nutrition and sometimes in clinical nutrition, maltodextrins resulting from industrial enzymic starch degradation are used, having a mixed content of glucose oligosaccharides, maltose, and glucose. It is often suggested that these maltodextrins are complex CHOs which result in a low and sustained glycemic response. However, there are no data to support this suggestion. In fact, the enzymic digestion of maltodextrins appears to take place at a high rate, which is also reflected by comparable post-ingestion insulin responses as well as oxidation rates during exercise compared with glucose [(32); Table 3].

**Table 3:** The glycemic index value of the plain carbohydrate tested vs. glucose as reference-control.

Glucose	GI−100
French baguette	GI−95
French fries	GI−75
Fructose, mean of three studies	GI−15
Macaroni, white boiled, mean of three studies	GI−50
Potato boiled, mean of seven studies	GI−53
Ripe banana, mean of nine studies	GI−48
Sourdough rye bread	GI−53
Spaghetti, white boiled. Mean of eight studies	GI−41
Sweet potato	GI−61
Sucrose	GI−67
White rice, mean of eight studies	GI−59
White wheat bread, mean of seven studies	GI−70
Whole grain rye bread, mean of four studies	GI−58

*Data Source: Atkinson et al. (18) and University of Sidney (33) online searchable data GI, International Tables of Glycemic Index and Glycemic Load Values*.

## Glycemic Index Classification and Its Meaning

The potential of CHOs to raise the level of blood glucose is often expressed as a glycemic index (GI) value. A high value refers to a strong elevation of blood glucose and is often seen as less healthy, whereas a low value is often seen as beneficial. When determining the GI value, glucose usually serves as the reference food with a glycemic index of 100. A food portion containing an amount of 50 g of available CHOs is ingested and the area under the blood glucose response curve is divided by the area resulting from the ingestion of 50 g of glucose. Full details on this matter can be found in Brouns et al. (34).

**Figure 3:**
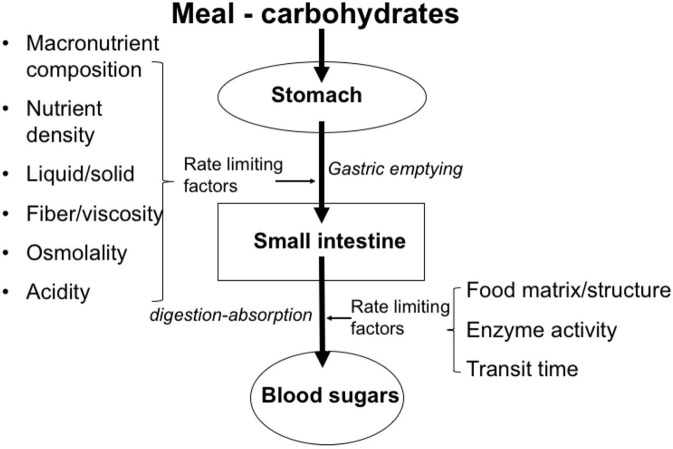
Factors that play a role in gastrointestinal transit, digestion, and absorption of saccharides.

Table 3 gives some examples of the glycemic index values of foods. It is important to understand that the glycemic index value in isolation cannot fully explain the physiological impact of CHO-based foods and beverages on health and disease. For example, the ingestion of 5 grams of glucose will not induce measurable hyperglycemia, despite its high GI value of 100. However, the ingestion of 50 grams will increase blood glucose very significantly. Thus, any GI value should be interpreted in the light of the quantity ingested. For this reason, the concept of the “glycemic load” of CHO-containing meals has been defined as a relevant approach. In addition, it needs to be noted that the GI value of any food prepared using these CHOs as a meal component is highly influenced by other factors that affect the rate of ingestion as well as the subsequent transit, digestion and absorption, see Figure 3. Examples are the content of enzyme inhibitors (e.g., α-amylase inhibitors) present in the CHO source, the overall macronutrient composition (quantity and type of CHO, fat, protein), the content and characteristics of dietary fibers (e.g., soluble, viscous, insoluble, bulking), the level of processing (e.g., level of refinement, such as the separation of bran and germ during milling, resulting in “refined” white flour), as well as the matrix effects (e.g., liquid vs. solid, starch in a compact elastic spaghetti structure vs. starch in a well-cooked soft potato). In the case of drinks, energy content and osmolality are factors which can significantly affect the gastric emptying rate as well as the related supply to the gut for absorption, depending on the concentration (35).

There is still one other point that needs to be addressed, especially related to sugars. The GI value of fructose (27) is very low and that of sucrose (36) is moderate. Thus, in terms of the viewpoint that a low to moderate GI is beneficial for health, one might conclude that fructose and sucrose are preferable for health to starches that have a much higher GI value. Based on current knowledge, this point is hard to substantiate. The suggestion that fructose is a single cause of non-alcoholic fatty liver disease driven by its dietary intake cannot be justified either based on data from excessive consumption (37).

The view that sugars added to beverages are a causal factor for obesity and diabetes is well-documented, because they cause a positive energy balance. However, in the case of sugars added to solid foods such as confectionery, this causal association has not been shown (2, 38–40). Data showing that two thirds of added sugars are being consumed in solid foods and only one third in beverages (41) raise questions about other factors that may play a role in addition to sugar (2, 3). From the above, it is clear that a focus on single CHO types, single CHO characteristics, or consumption in isolation as a single supply source has limited generalizability, especially when one wants to understand the overall effects of the diet containing these CHOs on postprandial appetite regulation, glycemia, lipidemia, low-grade inflammatory potential and possible health outcomes (42, 43). Moreover, the physiological status of the person in question also plays a significant role in how the human body manages the metabolism of saccharides. Elite endurance athletes such as professional cyclists ingest large amounts of refined carbohydrates, to a large extent in beverages, to maintain a high glucose availability for the benefit of delaying fatigue and maintaining a high-performance capacity. They burn the calories ingested, even when these exceed 6,500 kcal/day for 21 days (44), and accordingly do not become develop overweight. Based on these and other observations, their metabolism of the carbohydrates and the interrelationship with lipid metabolism will be quite different from that of inactive overweight individuals who are insulin-resistant or who suffer from type 2 diabetes when they consume large quantities of sugar-sweetened beverages [e.g., (45, 46)]. In this respect, it is obvious that specific food-based dietary guidelines are required for certain population subgroups.

## Legal and Who Definition of “Added Sugars” and “Free Sugars”

With respect to the classification and labeling of food and beverages, one should note that the term “sugars” on the food label generally stands for “*monosaccharides and disaccharides.”* In this respect, glucose and fructose are both simple sugars, but they behave very differently with regard to their metabolic effects. The hormonal responses that they induce (glucose is a significant driver of glycemia and insulin secretion, while fructose only has very minor effects on glycemia and insulin) and their metabolic fate, which includes the conversion to other intermediates such as organic acids (in particular lactic acid) as well as fatty acids, their use as fuel and their possible storage as glycogen or lipids differ. For this reason, it is important to have a basic understanding of the flow: type of carbohydrate → molecular characteristics → physiological aspects (digestion, absorption, and metabolic fate) → effects on health.

To give an example, oral glucose appears in blood as glucose and drives glycemia in a 1:1 ratio depending on the dose. Fructose, however, behaves differently because of its conversion to other metabolites and because of its very low insulinemic response (47). Although glucose and fructose are very often compared as monomers in metabolic studies, it needs to be addressed that humans usually do not consume fructose in isolation but almost always in combination with glucose, as it is present in sucrose- and HFCS-sweetened beverages, fruit juices, fruit syrups (see **Figure 5**), and fruits. Accordingly, the interpretation of data derived from studies in which fructose was supplied as monomer in high amounts should be seen in the light that this does not represent to normal human consumption situation. Concerns that all fructose from consumed SSBs and fruit juices goes straight to the liver where it is all converted to lipid are not supported by evidence. In contrast, most fructose is converted to non-lipid substrates.

**Figure 4:**
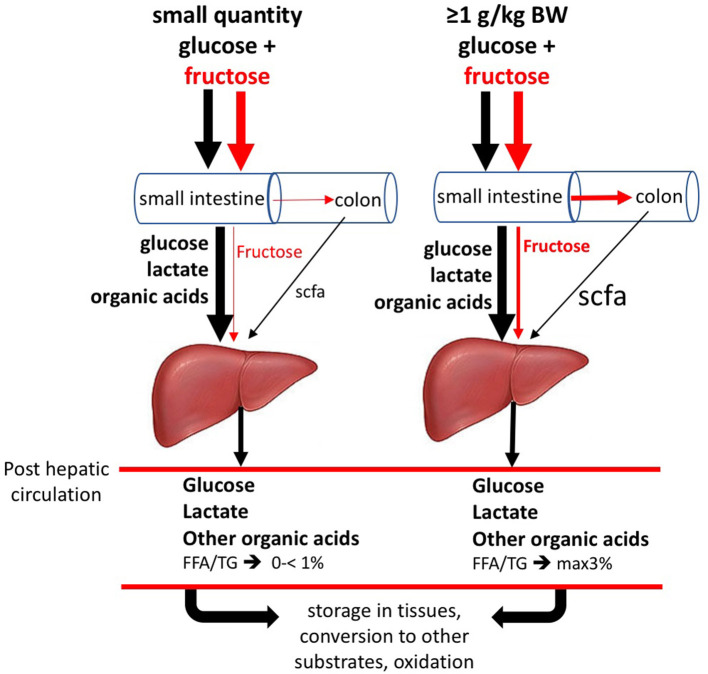
Metabolic fate of oral fructose. When ingesting small doses of fructose (F) and glucose together, as in human nutrition, most absorbed F is converted to glucose, lactic acid and other organic acids within the enterocytes, which appear in the portal vein supplying the liver. The amount of F passing to the liver after small oral doses is negligible. Glucose largely passes the liver and enters the blood circulation to be available to all tissues. Lactate will favorably be converted into liver glycogen. Non-converted lactate will pass on to the blood circulation. After ingesting acute large doses (≥1 g/kg body weight, equivalent to >1 liter of sugar-sweetened beverage/juice), F partly escapes its own slow absorption process and will pass on to the colon, where it may cause osmotic fluid shifts potentially leading to laxation and will be fermented by the microbiota leading to the formation of short-chain fatty acids, mostly acetate, which will be absorbed and pass on to the liver with portal blood. In this situation, the absorbed but non-converted fraction of F will serve as substrate for *de novo* fatty acids synthesis, along with the acetate coming from the colon. As a result of the above, F enters the circulation only in very small quantities. (Based on data from (12, 48–53)]. Figure based on data from Jang et al. (48) and Zhao et al. (53).

Recently Jang et al. (48) (Figure 4) performed double labeling studies allowing for quantitatively tracing the metabolic fate of fructose vs. glucose after supply to the mice. These researchers gave fructose together with glucose at 1:1 ratio, as normally is the case in human consumption of fructose containing saccharide sources. It needs to be noticed though, that for this work in mice, oral gavage by which the test dose was directly given into the stomach, was used. Using this procedure a large amount of fructose reaches the small intestine with much faster kinetics than typical human fructose consumption. However, while mouse metabolism is ~10 × faster than humans, rendering the faster fructose dose to metabolic rate ratio similar between the species (Jang, 2020 personal communication). Using this procedure, it was shown that a large fraction of the fructose absorbed in the small intestine is converted to glucose and organic acids within the enterocytes to such an extent that only very little fructose spills over to the liver. Thus, instead of the common perception that the liver is the prime fructose clearing-organ, it appears that small intestine fulfills this role. In case an acute high-dose of fructose saturates intestinal absorption and metabolic conversion capacity, a fraction on non-absorbed fructose partly passes from the small intestine to the colon, to be subsequently fermented by the microbiota giving rise to short chain fatty acids, mostly acetate, which will be absorbed and passed on the liver. The fraction of fructose that escapes metabolic conversion by the enterocytes also passes on the liver. Both acetate and fructose entering the liver can serve as a substrate for de novo triacylglycerol synthesis. The latter, however, remains relatively small, even in a situation of acute very high doses of fructose. Studies using stable isotopes in humans (1, 52) showed that the 3–6 h after ingestion high doses of fructose only a small percentage (<1%- max 3%) was converted to fatty acids. Thus, previous human work is in line with the new insights obtained by Jang et al. (48). Future studies in humans need to verify how much fructose, at real-life intake levels (concerning dose-time interrelationships generally much lower than experimental supply levels), really passes on the liver and the colon and what the conversion rate is to liver fat. To put this in perspective, early human studies, using the ileostomy model or breath hydrogen as marker of malabsorption, showed very clearly that fructose ingested as monomer at doses of >25 g induces malabsorption. However, when co-ingested with glucose (such as isomaltulose or sucrose)— even up to acute doses of up to 100 g sucrose (equivalent to about 1 l of SSB or fruit juice)— this is not the case (54–59). Since humans seldom consume fructose in isolation, this is an important point to consider. In addition, it needs to be addressed what other factors, apart from fructose contribute to the novo lipogenesis. In very recent work, it was shown that fructose fermentation derived acetate contributes to liver lipogenesis (53). Concerning the latter, a range of well-fermentable dietary fibers give rise to a significant amount of SCFA the cecum and colon, most importantly acetate, propionate, and butyrate, generally in a molar range of 70:20:10%, respectively. Individuals who consume relatively high amounts of dietary fiber such as fruit fibers and fructans (inulin) generally suffer less from being overweight. Why would fiber derived acetate, compared to fructose derived acetate, not or differently contribute to fatty liver? Is there a protecting role from propionate? (60). And, Why do physically active lean individuals, who consume substantial amounts of sugar, not suffer from an overweight and fatty liver, whereas most overweight individuals do? Is excess calories/positive energy balance the prime driving factor?

### Natural and Refined Sugars: Do They Differ?

**Figure 5:**
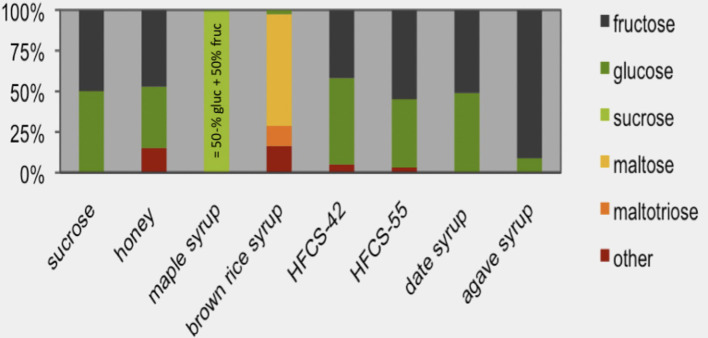
Sugars in syrups. The sugar monomer content of sucrose (sucrose water content is subtracted from the total mass and this value is set at 100%) is compared with high-fructose corn syrup (HFCS, containing either 42 or 55% fructose) and other types of syrups. Maple syrup consists almost entirely of sucrose [source: Andrea et al. (61)].

The metabolism of isolated monosaccharides and disaccharides (glucose, fructose, and sucrose/table sugar) is basically similar to that present in natural sources which contain mixtures of these sugars, such as in fruits or fruit-derived syrups. Because of their molecular similarity and related physiological responses, sugars naturally present in honey, fruit-derived syrups (Figure 5) and fruit juices have recently been proposed by the WHO (62) to be of similar nature as “commonly added sugars”. This approach has led to a new, mutually inclusive category of “free sugars” and to questions about the scientific basis of the term “free sugar”. For example, why are sugars in 100% fruit juice “free sugar” and the same sugars naturally present in the fruit not? Why is milk sugar naturally present in milk not considered to be a free sugar? In this respect, fruit juices have been classified in many epidemiological studies together with sugar-sweetened beverages (SSBs) as one category of “sugar-sweetened beverages”. Such a pooling of beverages and related observational data has resulted in the conclusion that fruit juices, similar to sucrose-sweetened drinks, are a cause of obesity. This outcome has led to international recommendations for reducing the consumption of “free sugar.” Table 4 gives an overview of definitions for “added sugars” used by various health authorities, as recently reviewed by Buyken et al. (63).

**Table 4:** Definitions of “added sugars” and their use in governmental reports [Source: Buyken et al. (63)].

“Sugars” are generally defined as “mono- and disaccharides.” Accordingly, “added sugars” is mostly considered to be “added mono- and disaccharides.” • WHO report (62): introduced the term “free sugars” as “all monosaccharides and di-saccharides added to foods by the manufacturer, cook, or consumer, plus sugars naturally present in honey, syrups, and fruit juices.” • US: United States Food and Drug Administration (US-FDA)-(64) and United States Department of Agriculture: Added Sugars are all sugars that are either added during the processing of foods, or are packaged as such, and these include sugars (free, mono- and disaccharides), syrups, naturally occurring sugars that are isolated from a whole food and concentrated so that sugar is the primary component (e.g., fruit juice concentrates), and other caloric sweeteners. • UK: SACN report (39) adopted the term “free sugars from WHO,” which now replaces the terms “added sugars” and “non- milk extrinsic sugars” (NMES) used previously. “Free sugars' comprises all monosaccharides^*^ and disaccharides^*^ added to foods by the manufacturer, cook or consumer, plus sugars naturally present in honey, syrups and unsweetened fruit juices. Under this definition, it includes lactose (the sugar in milk), when naturally present in milk and milk products, and the sugars contained within the cellular structure of foods (particularly fruits and vegetables) are excluded.” • EU: EFSA report (65): added sugars are “mono- and disaccharides and starch hydrolysates (e.g., glucose syrup, fructose syrup, maltodextrins) added during food preparation and manufacturing.”

Based on the molecular similarity of sugars, the pooling of juices and SSBs is understandable. However, data from intervention studies do not support this assumption. Murphy et al. (66) evaluated the effects of 100% fruit juice and measures of glucose control as well as insulin sensitivity in a systematic review and meta-analysis of randomized controlled trials. In this research, clinical trials were eligible for inclusion if the following criteria were met: [1] the trial was randomized and conducted in human subjects; [2] the trial was a controlled intervention providing 100% fruit juice and a control beverage (e.g., sugar/carbohydrate or energy-matched beverage, water or no beverage); [3] the fruit juice consumed was identified as 100% fruit juice; [4] subjects consumed 100% fruit juice for a minimum of 2 weeks; [5] outcome data for at least one measure of glucose control or insulin sensitivity were reported; and [6] reported outcomes included change from baseline values or baseline and endpoint values with error terms. It was concluded that the repeated intake of 100% fruit juice does not have a significant effect on glycemic control or measures of insulin resistance, which is consistent with findings from some observational studies in which the consumption of 100% fruit juice was studied separately from SSBs and in which lifestyle factors were also taken into account (2, 67). One reason may be that juice contains a wide variety of micronutrients and plant-bioactive substances from the original fruit, which may be “protective” (68). Another reason may be that individuals who decide to consume 100% juice instead of SSBs also make other healthy lifestyle decisions. As a result, 100% juice consumers usually have a more favorable body mass index [(69, 70); BMI], while the quality of the daily diet also appears to be better, as has been observed in both children and adults (68, 71). Very recently, Khan et al. (4) challenged the classification of juices in the same box as soda, since their consumption is associated with different health effects. This example also shows that looking at a single sugar type or sugar characteristic in isolation is not meaningful and may lead to wrong interpretations with respect to health.

Although the metabolism of CHO molecules naturally present in food or isolated (such as plain table sugar) is basically identical, it is important to understand as well that the food matrix can play a significant role in the rate of intake, digestion and absorption. The effects of sucrose added to a beverage (rapid gastric emptying and small intestinal absorption) will lead to a rapid increase in blood glucose and insulin, which differs from effects in a solid matrix such as confectionery (lower rate of digestion and absorption as well as a less rapid increase in blood glucose and insulin). As a consequence of its rapid gastrointestinal transit, sucrose in a beverage induces less satiation compared with sucrose in a solid food. This “incomplete sensing” drives “unnoticed” calorie intake, a positive energy balance and obesity, when happening frequently (72).

## Physicochemical, Technological, and Functional Characteristics Influence Sugars Metabolism

Sugars can also be listed according to their physicochemical, technological, and functional characteristics, which are important for food design and food processing (see Table 5). These characteristics can also affect the responses in our body. Two examples will be given here:

**Table 5:** Some physicochemical, technological, and functional characteristics that are important for food design and food processing.

• Sweetness • Solubility • Viscosity • Reducing power • Crystallinity • Glass transition temperature • Cooling effect (mouth) • Melting temperature • Freezing behavior

- The relative sweetness of sugars (Table 6) plays an essential role when sweetening foods and beverages. The lowest amount of a sugar needed to realize a certain sweetness is determined by the highest relative sweetness. Most used for sweetening is sucrose, the reason why the sweetness of sucrose is set at 100%. To replace sucrose (sweetness = 100) in a drink with glucose (in a concentration of 10% of its relative sweetness ≅ 70), 30% more glucose is required to achieve the same degree of sweetness. As a consequence, the beverage will contain more calories! To replace 100 g of sucrose in a beverage with fructose (relative sweetness of fructose at higher concentrations ≅ 150), 33% less sugar is required. As a consequence, the drink will contain less calories but also a high level of fructose, which may cause gastrointestinal distress/diarrhea and unfavorable metabolic effects.
- Glucose, fructose, galactose, lactose and maltose are reducing sugars. Sucrose and trehalose are non-reducing sugars. During the cooking/baking/roasting of food at high temperatures, reducing sugars react with amino acids in a Maillard reaction. This “browning reaction,” such as when baking meat or bread or roasting coffee, affects the taste and flavor. For this reason, selective use can be made of reducing sugars to obtain the desired browning and flavor (74). There is a wide range of Maillard reaction products (MRPs) known to influence digestive physiology, gut microbiota and metabolism, which are also suspected of triggering an immune reaction to and the allergenic potential of proteins (75). Overheating leads to the formation of advanced glycosylation end products (AGEs), which are thought to influence inflammation and possibly insulin resistance, whereas acrylamide (a product resulting from a reaction of a reducing sugar with the amino acid asparagine) is a known carcinogen. This information has prompted strategies to limit the formation of harmful MRPs. For example, limiting sugars as well as the asparagine content of potato and cereal products before thermal processing by measures such as selecting potato varieties with a low content of reducing sugars may help reduce acrylamide. Targeted potato storage temperatures such as storage below 8°C causes an increase in reducing sugar content and higher amounts of acrylamide. Modifying heat-processing conditions (time, temperature) and applying appropriate preheating treatments, such as soaking or blanching, can also help impact on the level of reducing sugars and thereby reduce the formation of MRPs (76, 77).

**Table 6:** Relative sweetness of sugars.

**Sugar**	**Relative sweetness**
**Monosaccharides**	
Fructose	115–180*
Glucose	50–70*
Galactose	54
**Disaccharides**	
Sucrose (gluc+fruc)	100
Maltose (gluc+gluc)	30–50*
Lactose (gluc+galac)	15–40*
Isomaltulose (gluc+fruc)	50
Trehalose (gluc+gluc)	45
HFCS–(gluc + 55% fruc)	>100
HFCS–(gluc + 42% fruc)	100

* *Degree of sweetness is influenced by concentration and higher at higher concentrations. Gluc, glucose; fruc, fructose; galac, galactose [Source: Clemens (73)]*.

## Sugars And Oral Health

Recently, the WHO (62) recommended to reduce the consumption of free sugars to preferably below 5% of the total energy intake as a conditional ^*^recommendation for both adults and children, the most important reason being the detrimental effects on oral health, despite the fact that the evidence was judged to be of a very low quality (62). [^*^Conditional recommendations are made when there is less certainty “about the balance between the benefits and harms or disadvantages of implementing a recommendation.” This means that “policy-making will require substantial debate and involvement of various stakeholders” for translating them into action (78)].

This evidence was based on data derived by experts as published in various reviews (79–82). Detrimental effects of sugars on oral health occur along two main routes: Firstly, this can be in the form of demineralization of enamel and dentine caused by acid, resulting from saccharolytic fermentation of sugars by oral microbiota; these monosaccharides and disaccharides include glucose derived from starch degradation by salivary amylase; Secondly, detrimental effects can result from exposure to food acids added to sugar-sweetened or light drinks, or acids naturally present such as in citrus juices, resulting in a low drink PH. These food-acids will directly erode the enamel and dentine without intervention of the oral microbiota In normal conditions, the acid present in the food/drink or formed by the microbiota is buffered over time and hence neutralized by saliva. In addition, saliva at neutral pH is supersaturated for calcium and phosphate, enabling the repair of the acid-induced demineralization (83). Acids derived from sugars can cause net demineralization when frequently taken and this is more detrimental if salivary buffer capacity is exceeded when saliva production is low or absent. Examples of the latter are athletes during intensive exercise when saliva production is inhibited and persons suffering from a low or absent salivary flow as a result of cancer radiation treatment, autoimmune diseases, (multiple) medications or physiologically by ageing.

Many studies have been performed to define the *in vivo* (in situ) cariogenic and erosive effects of sugars and acids on tooth mineral by the application of small intra-oral blocks of dentine or enamel or by using standardized solution enamel-rinsing essays *in vitro*. In the latter, the effects of remineralization can also be studied in detail. Depending on the frequency and dose, sucrose, glucose, fructose, lactose or starch may all result in demineralization (84–88). It appears that the molecular composition of sugars plays a role in the degree of fermentability by oral microbiota. For example, sucrose is composed of glucose and fructose, has an α-1,2 bond and is more rapidly fermented, and this lead to a critical lowering of plaque pH than isomaltulose, which is composed of the same monomers but which has an α-1,6 bond. Along similar lines, starch which is rapidly degradable by amylase and which leads to a higher glucose availability appears to be more cariogenic than slowly digestible starch which contains a higher fraction of amylose (89). It needs to be considered in this respect that a sticky food-matrix will increase tooth surface contact exposure time, thus enhance detrimental effects on tooth mineral. Sucrose is known to be most potent in causing cariogenicity, which raises questions; since the effects appear to be more potent than the effects of its composing monomers glucose and fructose.

Recently, it was hypothesized that an oral microbiota imbalance due to frequent sucrose exposure may be a causal factor driving sucrose to be more harmful because sucrose exposure disrupted the homeostasis between acid-producing and alkali-producing bacteria (90). Because the oral microbial composition and metabolism changed significantly with sucrose exposure, while no significant difference was detected after lactose and glucose exposure, the authors claim that these findings indicate that the cariogenicity of sugars is closely related to their effects on the oral microecology.

Acidified drinks containing substantial amounts of sucrose are of particular concern (91, 92), because they do not only cause caries but also dental erosion. Even acidified drinks with low sugar contents or without sugars making use of non-sugar sweeteners are erosive because of the acids present therein result (93). Despite the primary focus on the role of sugars in causing caries, it should be noted that the process of dental erosion and caries initiation is multifactorial (36, 94).

In particular, the effects of sucrose appear to be of great concern during childhood, given the fact that SSBs intake significantly increases the caries burden in 10-year-olds with attenuated effects in 15-year-olds-age groups that are known to be the highest consumers of free sugars. To prevent caries, SSBs consumption should therefore be reduced, especially in children and adolescents (95). Of great concern are a simultaneous combination of high sipping frequency and low PH beverage and sugar concentration, especially in young children, leading to early childhood caries. Giving very young children sugary drinks in a sipping bottle will lead to continuous small quantities flushing especially of the front teeth. This process will be even more detrimental if the child falls asleep, resulting in a low salivary flow and the reduction of the salivary-buffering effect (96, 97). There is no doubt about the fact that sugar and food acids are not the only factors of importance. Poor oral hygiene, use of fluoride, appropriate salivary flow, presence of calcium in the drink/food, type of food acid used (94), consumption pattern and bottle or breastfeeding (97) play a role in the etiology of caries. In addition, the frequency of exposure may be more relevant than the quantity. Van Loveren (98) addressed the question of which sugar-reducing strategy is the best for caries prevention. To answer this question, the following aspects should be addressed: the shape of the dose-response association between sugar intake and caries, the influence of fluoridated toothpaste on the association of sugar intake and caries, as well as the relative contribution of frequency and amount of sugar intake to caries levels. The author argues that when fluoride is appropriately used, the relation between sugar consumption and caries is very low or absent. The high correlation between amount and frequency hampers the decision on which of the two is more important. Reducing the amount without reducing the frequency does not seem to be an effective approach to prevent caries.

## Concluding Remarks

All rapid fermentable sugars give rise to acid production by microbiota present in the oral cavity which, dependent on frequency of exposure, salivary buffer capacity, presence of calcium for remineralization and oral hygiene status will impact on erosive potential and cariogenicity. All digestible CHOs deliver “sugars” as monosaccharides to the gut epithelium for absorption. Post-absorption, the metabolism of these monomers is basically identical and independent of the original source. However, the way in which CHOs have been processed (natural, low-processed vs. refined/highly processed, and heat-exposed), the matrix in which these CHOs are present (e.g., liquid, solid, viscous, and non-viscous), the co-presence of other nutrients (e.g., proteins, polyphenols, vitamins, minerals, and plant-bioactive substances) in the natural CHO source/matrix vs. their absence in refined CHOs and the dose ingested all play a role in the overall effects in the human body. Looking at one particular CHO characteristic will almost always lead to a different conclusion, such as that fructose is toxic (99) than evaluating from a “total perspective”; fructose is only toxic at excessive exposure levels that do not mimic human consumption (1, 3). It appears that mutual and interactive effects exceed the sum of the individual characteristics, while they also determine the effects on health and disease. For this reason, an increased focus on the overall effects and quality of carbohydrate sources and meals for food-based guidelines rather than individual component-based recommendations is desired.

## Author Contributions

The author confirms being the sole contributor of this work and has approved it for publication.

## Conflict of Interest

The author declares that the research was conducted in the absence of any commercial or financial relationships that could be construed as a potential conflict of interest.

## Abbreviations

AGEs: Advanced glycosylation end-products
CHO: carbohydrate
CHOs: : carbohydrates
DP: degree of polymerization
GI: glycemic index
MRPs: Maillard reaction products
SCFAs: short chain fatty acids
SSBs: sugar sweetened beverages.
